# Supplementary material for: Association between urinary N-acetyl-β-glucosaminidase activity–urinary creatinine concentration ratio and risk of disability and all-cause mortality
Source: PLoS One. 2022 Mar 25;17(3):e0265637. doi: 10.1371/journal.pone.0265637 (PMC8956177; doi:10.1371/journal.pone.0265637)
Supplement: S1 File — (DOC) [file pone.0265637.s001.doc]

November 30 2005

Iwao Kaneko, Director, Hidaka Medical Center

Shin-ichiro Tanaka, Head of Internal Medicine

**Patients Who Underwent Cardiovascular Disease**

**Preventive Screening and Their Family Members**

**(Request for assistance with additional follow-up questionnaire)**

Thank you for your cooperation with the follow-up survey on Cardiovascular Disease Preventive Screening last year. We are currently collating the questionnaire findings and within this fiscal year will summarize the results and reflect them in health maintenance activities for our patients.

In the survey carried out last year, we asked about diseases; as the present status of those with the same disease may vary from actively working to being bed-ridden we currently plan to conduct a survey in order to investigate the degree of disability in people’s daily lives due to them. If a patient is certified to receive long-term care, we would also like to ask the level of care as a reference for the survey.

For this purpose, we would be grateful for your assistance by completing the enclosed questionnaire and then returning it in the enclosed envelope.

At this busy time at the end of the year, we really appreciate your cooperation.

If it is difficult for a patient receiving long-term care to fill out the questionnaire themselves, we would be grateful if a family member could fill it out for them. We would be much obliged if the questionnaire could be returned by the end of December.

(Among 2,155 persons who underwent preventive screening for cardiovascular diseases in 1993, this questionnaire is being sent to those who cooperated with last year’s follow-up survey and are currently 65 years or older.)

**Screening Questionnaire concerning Long-Term Care service use Part 1**

Name: Consultation No.:

Name in Roman alphabet: Date of birth:

**Question 1.** Please state the patient’s present status.

　　　1) Living　　　2) Deceased (Date of death: (mm) (dd) (yy))

　　　　For those who replied living:

**Question 2.** Are you currently certified for long-term care?

　　　1)　Yes　　　2) No

　　　　This is the end of the questionnaire for those who replied No.

For those who replied Yes:

**Question 3.** Please indicate your current care requirement level by circling one of those

below.

(For care level, please refer to the enclosed sample long-term care insurance

certificate)

Need support　Care level 1　Care level 2　Care level 3　Care level 4　Care level 5

**Question 4．**Finally, please tell us who completed the questionnaire.

　1) Patient themselves

2) Other than patient (Relationship 　　　　　　　　　　　)

Thank you for your cooperation.

Please also see the next page.

**Consent Form**

To Director of Hidaka Medical Center:

Purpose of survey: To investigate disease status and long-term care status through further follow-up survey of those who underwent cardiovascular disease preventive screening

For the above purpose, I consent to the statement of the attending physician for long-term care insurance certification being read.

Date (mm, dd) 20___

Address: ___ ____Hidaka-Cho Toyooka City

Name (　　　　　　　　　　　　　　　　　)

（Name of proxy　　　　　　　　　　　　　　　　）　　Seal

Thank you for your cooperation
