# Supplementary material for: Association between urinary N-acetyl-β-glucosaminidase activity–urinary creatinine concentration ratio and risk of disability and all-cause mortality
Source: PLoS One. 2022 Mar 25;17(3):e0265637. doi: 10.1371/journal.pone.0265637 (PMC8956177; doi:10.1371/journal.pone.0265637)
Supplement: S2 File — (DOCX) [file pone.0265637.s002.docx]

**Screening Questionnaire concerning Long-Term Care service use Part 2**

Name Consultation No. at time of examination:

Name in Roman alphabet: Date of birth:

Address: Tel. No.

This questionnaire asks about the status as of 1993 of a person certified for long-term care at present.

If the person themselves cannot respond, for instance if they have passed away, we would be grateful if a family member could reply for them.

Was long-term care required in1993? (Please choose either of the following)

Yes　　No

If the answer is no, the questionnaire is over.

The following are questions for those who answered “Yes”　　 Remarks

Please tell us the status of the recipient of care then.

Physical status of care recipient in 1993 (Please select one of the following)

- Could go out by myself
- Independent at home but care necessary when going out.
- Care needed for daily living at home
- Bedridden

Confirmation of dementia care status in 1993　(Please select one of the following)

- Had dementia but was largely independent
- Mild (got lost, could not pay for things, etc.)
- Moderate (Needed help with changing clothes, eating or going to toilet)
- Severe (had to be watched constantly)
- Significant psychiatric symptoms and problematic behavior; specialized treatment required

This is the end of the questionnaire

Thank you for your cooperation.
