# Supplementary material for: Association between urinary N-acetyl-β-glucosaminidase activity–urinary creatinine concentration ratio and risk of disability and all-cause mortality
Source: PLoS One. 2022 Mar 25;17(3):e0265637. doi: 10.1371/journal.pone.0265637 (PMC8956177; doi:10.1371/journal.pone.0265637)
Supplement: Attachment — Submitted filename: Response to Reviewers_for submission_2021_12_27.docx [file pone.0265637.s004.docx]

PONE-D-21-27646

Urinary N-acetyl-β-glucosaminidase activity–urinary creatinine concentration ratio predicts risk of disability or early death

Shin-ichiro Tanaka et al

Journal Requirements:

When submitting your revision, we need you to address these additional requirements.

1. Please ensure that your manuscript meets PLOS ONE's style requirements, including those for file naming. The PLOS ONE style templates can be found at 
https://journals.plos.org/plosone/s/file?id=wjVg/PLOSOne_formatting_sample_main_body.pdf and 
https://journals.plos.org/plosone/s/file?id=ba62/PLOSOne_formatting_sample_title_authors_affiliations.pdf

2. Please upload an English language copy of the questionnaire as a supplementary file.

3. In your Data Availability statement, you have not specified where the minimal data set underlying the results described in your manuscript can be found. PLOS defines a study's minimal data set as the underlying data used to reach the conclusions drawn in the manuscript and any additional data required to replicate the reported study findings in their entirety. All PLOS journals require that the minimal data set be made fully available. For more information about our data policy, please see http://journals.plos.org/plosone/s/data-availability.

Upon re-submitting your revised manuscript, please upload your study’s minimal underlying data set as either Supporting Information files or to a stable, public repository and include the relevant URLs, DOIs, or accession numbers within your revised cover letter. For a list of acceptable repositories, please see http://journals.plos.org/plosone/s/data-availability#loc-recommended-repositories. Any potentially identifying patient information must be fully anonymized.

Important: If there are ethical or legal restrictions to sharing your data publicly, please explain these restrictions in detail. Please see our guidelines for more information on what we consider unacceptable restrictions to publicly sharing data: http://journals.plos.org/plosone/s/data-availability#loc-unacceptable-data-access-restrictions. Note that it is not acceptable for the authors to be the sole named individuals responsible for ensuring data access.

We will update your Data Availability statement to reflect the information you provide in your cover letter.

4. Your ethics statement should only appear in the Methods section of your manuscript. If your ethics statement is written in any section besides the Methods, please delete it from any other section.

5. Please include captions for your Supporting Information files at the end of your manuscript, and update any in-text citations to match accordingly. Please see our Supporting Information guidelines for more information: http://journals.plos.org/plosone/s/supporting-information.

[Note: HTML markup is below. Please do not edit.]

**Response**

We have addressed the above requests and made necessary changes. The English versions of questionnaires are now included as Supporting Information with captions presented at the end of manuscript. Regarding data availability, data cannot be shared publicly because they include potentially identifying or sensitive information about the study participants. However, they will be shared on reasonable request. This is now explained in the data availability statement section.

Reviewers' comments:

Reviewer's Responses to Questions

**Comments to the Author**

1. Is the manuscript technically sound, and do the data support the conclusions?

The manuscript must describe a technically sound piece of scientific research with data that supports the conclusions. Experiments must have been conducted rigorously, with appropriate controls, replication, and sample sizes. The conclusions must be drawn appropriately based on the data presented.

Reviewer #1: Partly

Reviewer #2: Partly

Reviewer #3: No

2. Has the statistical analysis been performed appropriately and rigorously?

Reviewer #1: Yes

Reviewer #2: No

Reviewer #3: No

3. Have the authors made all data underlying the findings in their manuscript fully available?

The PLOS Data policy requires authors to make all data underlying the findings described in their manuscript fully available without restriction, with rare exception (please refer to the Data Availability Statement in the manuscript PDF file). The data should be provided as part of the manuscript or its supporting information, or deposited to a public repository. For example, in addition to summary statistics, the data points behind means, medians and variance measures should be available. If there are restrictions on publicly sharing data—e.g. participant privacy or use of data from a third party—those must be specified.

Reviewer #1: Yes

Reviewer #2: No

Reviewer #3: No

4. Is the manuscript presented in an intelligible fashion and written in standard English?

PLOS ONE does not copyedit accepted manuscripts, so the language in submitted articles must be clear, correct, and unambiguous. Any typographical or grammatical errors should be corrected at revision, so please note any specific errors here.

Reviewer #1: Yes

Reviewer #2: Yes

Reviewer #3: Yes

5. Review Comments to the Author

Please use the space provided to explain your answers to the questions above. You may also include additional comments for the author, including concerns about dual publication, research ethics, or publication ethics. (Please upload your review as an attachment if it exceeds 20,000 characters)

Reviewer #1: The authors evaluated whether increased urinary N-acetyl-β-glucosaminidase activity to creatinine ratio (NCR) sampled from spot urine was predictive of disability or death in a total of 1,209 elderly Japanese. The authors performed multivariable logistic regression and found that subjects with the highest quartile of NCR had increased risk of disability, death, and combination of the two.

Overall, the paper contained valid information on NCR and adverse outcome. However, there are several significant issues that need to be addressed.


Major comments

My first major concern is about the description of the study, which is different from previously published papers (reference 19-21).
1. The baseline of the current study, unlike the what the authors wrote in the abstract, the methods and the results that "this study started in December 2005", was collected at year 1993.

**Response**

Thank you for pointing this out. We have specified the observation period in the results section of the abstract as follows: ‘A total of 1182 participants were followed up for a median of 12.4 years’ (line 30).

2. Follow-up was conducted more than ten years later, around 2005. The endpoints including disability and death were collected at this period.
The authors should 1) clarify the exact inclusion criteria, e.g., aged >= 65 years at the time of follow-up. 2) correct all related sentences and provide the duration of the follow-up. 3) conduct composite outcome including all-cause mortality and disability, when exact date of disability was possible. 4) modify the study flow chart accordingly (figure 1).

**Response**

1. The inclusion criterion is now clarified in the *Study population* subsection of *Participants and Methods*: ‘The inclusion criterion for the present study was age ≥65 years at initiation of the follow-up study at the end of October 2005’ (lines 96–97).
2. The dates of the baseline survey and follow-up study are now specified in the *Data collection and laboratory tests* and *The follow-up study* subsections, respectively, of *Participants and Methods*: ‘The baseline survey was conducted between July and August 1993’ (line 130) and ‘Completed questionnaires were collected between January and March 2006’ (line 155-156). The follow-up periods are also specified: ‘Therefore, the mean and median follow-up periods for total study population were 11.4 years and 12.4 years, respectively’ (lines 156–157).
3. Participants were not asked their exact date of entry into the LTC insurance program. Therefore, the Cox proportional hazards regression model could not be used to estimate the risk of disability. Instead, we conducted an additional analysis using another Cox regression model to investigate the relation between baseline urinary NAG/creatinine ratio and risk of all-cause mortality. This is explained in the *Statistical analyses* subsection thus: ‘To estimate risk of disability, we used the multiple logistic regression model rather than the Cox proportional hazards model, because data on the exact date on which participants entered the public LTC insurance program had not been collected.’ (lines 169-171).
4. The participant selection flow chart (Fig 1) has been modified accordingly.

Secondly, given the long-term care act was implemented around year 2000, and that the disability status could not be determined at baseline ("apparently healthy"), the conclusion that NCR PREDICTED risk of disability could not be drawn. Unless additional information was provided, baseline NCR was ASSOCIATED with disability in the cross-sectional survey conducted at follow-up. Meanwhile, were all subjects screened for eligibility of LTC or did they need to apply for it?

**Response**

We agree with your point and have therefore added information to the analysis. At the baseline survey, we identified participants who were bedridden, and in the follow-up study, we asked participants if they had needed nursing care at the time of the baseline study. Participants who were bedridden or needed nursing care at baseline have been excluded from the analysis. Additionally, we have added baseline physical activity to the model as a covariate, to adjust for potentially underlying disability and disease in participants with low physical activity (‘Physical activity is another confounder, because participants with low daily activity could have potential disability or disease...’; lines 178–180).

As explained in the *Limitations* subsection of the *Discussion*, ‘we did not collect data on participants’ disability status but simply asked if they were using the public LTC insurance program’ (lines 422–423); their disability status was confirmed by officials who visited them at home. ‘Therefore, participants with a disability but who did not use the service might not have been recognized as having a disability. This may have contributed to reducing the strength of the association between baseline urinary NAG/creatinine ratio and risk of disability in this study.’ (lines 423–426). It is possible that such participants did not need any government support.

Thirdly, additional statistical analysis concern should be addressed apart from the Cox regression as mentioned in the first major comment.
1. The ranges for NCR quartiles, numbers and rates of events should be provided.

**Response**

We have added baseline characteristic data for urinary NAG–creatinine ratio quartiles to Table 2. We have also presented the numbers of events in Table 1 and the *Results* (‘A total of 830 (70.2%) were not receiving LTC and were not reported to have died; they are subsequently reported as the no disability and alive (*NA*) group. Of the remainder, 122 (10.3%) were receiving LTC and 230 (19.5%) were reported to have died.’; lines 200–202).

2. The Relative Risk should be Odds Ratio in logistic regression. A raw calculation from the data provided in the supplemental file also confirmed the need to replace the term RR with OR.

**Response**

Thank you for this information, which is important for the presentation of the data. We have changed the relevant terms as advised.

3. Analysis for continuous NCR should be conducted.

**Response**

We have added the results of multivariable adjusted analyses for continuous NCR to Tables 4, 5, and 6. In each case, NCR was significantly associated with both outcomes.

4. Re-classification of predicted risk after adding the NCR into base model should be analyzed, even if non-significant. This is particularly important given the fact that the age of subjects in DD group on average was 10 years older than NA group and reached the healthy life expectancy of 74.1 years.

**Response**

Thank you for your important point about the analyses.

We have included all variables in the multivariable-adjusted models that we used in the current study, and we have described every predicted risk for each variable in Table 6.

Fourthly, introduction and discussion section should be revised to give readers a clear and objective view on the current finding.
1. The population included in the current study were not all CKD patients, indeed most of them were free of CKD. First paragraph of introduction focused on CKD, which is not the population, nor the disease need to diagnose in the population. The authors may, for instance, give a summary of kidney biomarkers and population risk here.

**Response**

Thank you for your helpful comment.

In the *Introduction*, we refer to the prevalence of CKD in the Japanese population (13% of persons aged >20 years). Additionally, we give the prevalence of CKD (defined as eGFR <60 mL/min/1.73 m^2^) in our cohort: 22.4% (see Table 1). We also refer to previous reports that high urinary NAG activity is associated with the risk of cardiovascular disease, heart failure, and mortality (‘High urinary NAG activity has been reported to be associated with future risk of all-cause mortality and hospitalization in patients with heart failure, and measuring urinary NAG activity in addition to eGFR and urinary albumin excretion has been shown to improve prediction of these events [15]. In a population study, urinary NAG/creatinine ratio has also been shown to be associated with risk of myocardial infarction, ischemic stroke, and all-cause mortality, independently of urinary albumin excretion and cardiovascular risk factors [16].’; lines 67–72).

2. Details on the act of long-term care and its use in the determination of disability should be put into methods section. Disability, on the other hand, could be discussed here as a general term.

**Response**

Thank you for this helpful comment. In the *Participants and methods* section, we now state how we have defined disability in the *Study population* subsection (‘Recipients of support from this program are considered to have age-related impairment and were therefore defined in this study as having a disability’; lines 99–101) and provide details of long-term care in the *Exposure and outcomes* subsection (lines 114–126).

3. The conclusion in the first paragraph of discussion that subjects with proteinuria could not benefit from NCR test is underpowered given there were only 56 subjects with proteinuria. The conclusion therefore should be downplayed.

**Response**

In accordance with your recommendation, we have downplayed this statement in the first paragraph of the *Discussion* (‘This relation remained significant even in participants without proteinuria.’; lines 375–376*)*.

4. The comparison of current study with existing evidence on NCR and adverse outcome should be discussed firstly. Multiple studies focused on this topic could be retrieved from PubMed search, e.g., the paper by Solbu et al. published in J Am Soc Nephrol (27: 533–542, 2016, doi: 10.1681/ASN.2014100960), compared NCR with albumin-to-creatinine ratio in risk prediction of mortality in a low-risk population.

**Response**

In the *Introduction*, we refer to previous reports that focused on the relation between NCR and risk of cardiovascular diseases and mortality (lines 67–80). However, we are willing to move this information to the *Discussion*, if advised to do so.

5. The relationship among NCR/CKD, aging and cardiovascular risk should be clearly illustrated. In the second paragraph of discussion the authors made a bold statement that CKD is related to physiological change of aging rather than that of CVD, which is not appropriate as in the following paragraph the authors explained mechanisms involving in such procedure, i.e., lack of BP control, whereas hypertension is one of the most important CV risk factors.

**Response**

We refer to nocturnal hypertension as an example of ‘masked hypertension’. People with this condition may not be recognized as having hypertension in epidemiological studies based on office hypertension, leading to misclassification of the disease (lines 382–388).

We did not perform ambulatory blood pressure monitoring in this study, so we were unable to confirm this hypothesis.

6. Is there any interplay between NCR and Klotho and FGF23? Please provide pathophysiological and/or epidemiological links here given the current study population was only general but not CKD population. Alternatively, replace them with other evidence on the pathophysiological importance of NCR.

**Response**

NCR simply represents tubular and interstitial injury of the kidney. We were unable to find any interactions between NCR and FGF23 or Klotho. However, all of them relate to kidney function, and considerable increases in NCR and FGF23 and a decrease in Klotho in relation to decreases in kidney function have already been reported.

In addition to studies using experimental animal models, we found a number of epidemiological studies regarding relations between Klotho and FGF23 and the future risk of cardiovascular disease, death, and frailty in the general population. We have added this information to the *Discussion* (lines 392–401).

7. Given the current guidelines from KDIGO and other organizations all recommend the use of ACR and eGFR in CKD staging, whether NCR levels were indicative of CKD stage should be addressed before further discussion on CKD.

**Response**

In the *Discussion*, we propose using additional kidney injury markers alongside eGFR and ACR to achieve improved estimates of future risk of disability and death in the general population.

8. eGFR is an established risk marker of CKD but it should be used in conjunction with others when eGFR is not low (<60ml/min/1.73m2). Current data suggested the population included here is not particularly of impaired renal filtration. Relevant discussion on eGFR in comparison to NCR should be shortened and organized.

**Response**

Thank you for this insight.

Because eGFR is already an established risk marker, we have reduced discussion of it.

Minor comments
To make it less confusing, only keep the number of included subjects in the abstract and delete the total number screened.

**Response**

As suggested, the number in the baseline population has been deleted from the abstract; only the number of participants is specified (line 30).

Line 127 of page 6, "an" MCP should be "a" MCP.

**Response**

The text has been changed as requested.

In the methods section, please specify the name and version of statistical software.

**Response**

The statistical software is now specified: ‘SPSS 11.01J software for Windows (SPSS, Japan, Tokyo, Japan) was used to perform all statistical analyses.’ (lines 190–191).

In the methods section, please specify the regression models used for the evaluation.

**Response**

In the *Statistical analyses* subsection, we now explain why the logistic regression model was chosen for estimating disability: ‘To estimate risk of disability, we used the multiple logistic regression model rather than the Cox proportional hazards model, because data on the exact date on which participants entered the public LTC insurance program had not been collected. Furthermore, this program was implemented from April 2000 and the baseline survey was conducted in 1993; the LTC service was not available between 1993 and March 2000’ (lines 169–173).

We also used the Cox proportional hazards regression model to estimate the risk of all-cause mortality (please see our response to Reviewer #1’s second comment).

Table 4 has duplicated results with Table 3 (the "entire cohort"). Similarly Figure 2 is a graphical presentation of some results presented in Table 3. Such items should be removed.

**Response**

We have deleted the results for the entire cohort from the multivariable analysis results presented in Table 4. We have also deleted Figure 2.

Conclusion "public LTC insurance services" should be replaced by disability or disability determined by public LTC insurance services.

**Response**

As requested, ‘subsequent need for public LTC insurance services’ has been replaced by ‘disability’.

Reviewer #2: In this manuscript, the author has demonstrated that the activity of urinary N-acetyl-β-glucosaminidase (NAG), a marker of kidney injury, is associated with subsequent risk of disability or early death in a general population. However, there exists several problems.
1、 What is the specific date on which to determine whether the events occurred or not？Please describe the medium and IQR of the follow-up period.

**Response**

The follow-up period is now mentioned in the abstract (‘A total of 1182 participants were followed up for a median of 12.4 years.’; line 30) and *Participants and Methods* (‘the mean and median follow-up periods were 11.4 years and 12.4 years, respectively’; lines 156–157).

2、 The association between the quartile groups of NAG groups and disability or death should be evaluated using the Kaplan-Meier survival method and compared using log-rank statistics.

**Response**

Participants were not asked their exact date of entry into the LTC insurance program. Therefore, we were unable to use the Kaplan–Meier survival method to estimate the incidence of disability. Instead, we used the model to estimate all-cause mortality. However, the results were equal to those of the univariate analysis obtained using the Cox proportional hazards model.

If our study had been a randomized controlled trial, it would have been helpful to present the cumulative survival rate in figure form, because all confounders had already been adjusted for at the time of randomization. However, the cohort study results require adjustment for potential confounders; simply presenting the results of univariate analysis in figure form may lead to misunderstanding of the results.

3、 How about the relationships between urinary NAG and other clinical variables?

**Response**

Thank you for making this important point. To confirm collinearity, we performed a correlation analysis between urinary NAG and other clinical variables (see Table 3).

4、 The association between the NAG and cardiovascular mortality and renal mortality should better be evaluated.

**Response**

We did not set end-stage renal failure as an endpoint. Therefore, we were unable to determine whether a high urinary NAG to creatinine ratio was associated with increased risk of end-stage renal failure.

We have previously used the Cox proportional hazards model to investigate the relation between urinary NAG activity and cardiovascular events. However, we found no association between increased urinary NAG activity and risk of cardiovascular disease.

5、 What is your criteria for selecting independent variables in your multivariable-adjusted model？

**Response**

We included traditional cardiovascular risk factors as covariates, because most cardiovascular risk factors are also risk factors for CKD. Furthermore, to adjust for potentially underlying disability and diseases among the participants, we also used physical activity as a covariate (lines 174–183).

6、 The usage of anti-hypertensive drugs, anti-diabetic drug, statins and comorbid conditions should be recorded and adjusted.

**Response**

Unfortunately, participants were not asked whether they had used these drugs during the follow-up period. Therefore, we are unable to adjust the analyses for these confounders.

7、 In order to determine whether risk prediction models were improved by addition of the NAG, C-index should be calculated for the demographic, eGFR, and cardiovascular risk factor model for each outcome.

**Response**

Thank you for your helpful suggestion. It would be useful if we could create a new model for predicting future disability. However, the aim of this paper was to establish an association between NAG/creatinine ratio and disability or early death. Creation of a new model for predicting future disability is beyond the scope of this paper. However, we will try to do this in the future.

8、 Q1 needs to be placed in the table, and the specific values of Q1-Q4 need to be marked.

**Response**

Thank you for your helpful comment. We have made the changes requested.

9、 NAG is one of the urine kidney injury biomarkers, not equals to CKD. These two concepts should not be confused.

**Response**

Thank you for this observation.

We regard NAG activity as a kidney injury marker and CKD as a consequence of chronic kidney injury.

10、 In line 277-279, you mentioned that “The association between urinary NAG/creatinine ratio and disability or death, which was found to be independent of cardiovascular risk factors (including age), suggests that CKD is related to the physiological changes of aging rather than those of cardiovascular disease. ”, while I am so confused about this sentence. How can I come to a conclusion that “CKD is related to the physiological changes of aging” from your data? The discussion should be re-arranged.

**Response**

We agree that this statement was confusing and an overstatement. We have replaced it with discussion about the mechanisms underlying CKD and aging, with reference to several relevant studies (lines 392–401).

11、 In your opinion, what is the probably reason that urinary NAG/creatinine ratio can not predict subsequent disability or death in patients with proteinuria?

**Response**

As mentioned in the *Limitations* subsection, ‘the great majority of the total cohort did not have proteinuria’ (lines 431–432); we considered the number of participants with proteinuria (n=56) to be too low to allow estimation of the risk of disability or death.

12、 I think Table 2 and Figure 2 maybe not necessary.

**Response**

Thank you for your comment. We have deleted Figure 2 and amended Table 2.

Reviewer #3: The description of the study design in this article is incomplete and lacks specific follow-up methods. There are also doubts about the statistical methods used in this article to illustrate the relationship between urinary NAG/creatinine ratio and the risk of death and disability. So I don't think this article has reached the standard for receiving manuscripts. Questions are as follows:
1. What is the collection method of the outcome event data? At what time were the collection points?

**Response**

We mailed the questionnaires to the participants or their relatives at the end of December 2005 and collected outcome data between January and March 2006. For details, please see the subsection *The follow-up study* of *Participants and Methods*; lines 153–159).

2. Since the beginning of the study in 2005, what was the median follow-up time for all participants? How many subjects were lost to follow-up?

**Response**

The median follow-up time was 12.4 years (see Table 1).

A total of 1438 participants who were aged ≥ 65 years at the initiation of the follow-up study at the end of October 2005 were eligible for this study. Only 45 subjects were lost to follow-up (see *Results*; line 198). Initially, we collected 1400 responses. However, during the Cox hazards regression analysis, we found several faults in the responses (e.g. no descriptions of the date of death). Therefore, 1393 completed questionnaires were used.

3. The statistical method used in the analysis of survival data in this paper used multivariate logistic regression analysis instead of the Cox risk regression model (Table 2 and Table 3), which ignored the impact of censored data.

**Response**

Thank you for your comment.

We did not collect data on the exact date of participants’ entry into the public LTC program. Therefore, we were unable to use the Cox regression model. However, if participants were able to discontinue their use of the service, they may be considered to no longer have a disability.

For data from participants who had died, it is preferable to use the Cox regression model. Therefore, in the revised manuscript, we describe how we used the Cox proportional regression model to estimate the relation between baseline urinary NAG/creatinine ratio and future risk of all-cause mortality.

4. In Table 4, participants with proteinuria were only 56, which could produce an unstable model.

**Response**

We agree and have revised the manuscript to present the results for risk in participants *without* proteinuria only.

6. PLOS authors have the option to publish the peer review history of their article (what does this mean?). If published, this will include your full peer review and any attached files.


If you choose “no”, your identity will remain anonymous but your review may still be made public.


**Do you want your identity to be public for this peer review?** For information about this choice, including consent withdrawal, please see our Privacy Policy.

Reviewer #1: No

Reviewer #2: No

Reviewer #3: No

[NOTE: If reviewer comments were submitted as an attachment file, they will be attached to this email and accessible via the submission site. Please log into your account, locate the manuscript record, and check for the action link "View Attachments". If this link does not appear, there are no attachment files.]

While revising your submission, please upload your figure files to the Preflight Analysis and Conversion Engine (PACE) digital diagnostic tool, https://pacev2.apexcovantage.com/. PACE helps ensure that figures meet PLOS requirements. To use PACE, you must first register as a user. Registration is free. Then, login and navigate to the UPLOAD tab, where you will find detailed instructions on how to use the tool. If you encounter any issues or have any questions when using PACE, please email PLOS at figures@plos.org. Please note that Supporting Information files do not need this step.
