# Supplementary material for: Association between urinary N-acetyl-β-glucosaminidase activity–urinary creatinine concentration ratio and risk of disability and all-cause mortality
Source: PLoS One. 2022 Mar 25;17(3):e0265637. doi: 10.1371/journal.pone.0265637 (PMC8956177; doi:10.1371/journal.pone.0265637)
Supplement: Attachment — Submitted filename: Reviewers comments_2022_02_24.docx [file pone.0265637.s005.docx]

Reviewers' comments:

Reviewer's Responses to Questions

**Comments to the Author**

1. If the authors have adequately addressed your comments raised in a previous round of review and you feel that this manuscript is now acceptable for publication, you may indicate that here to bypass the “Comments to the Author” section, enter your conflict of interest statement in the “Confidential to Editor” section, and submit your "Accept" recommendation.

Reviewer #1: All comments have been addressed

Reviewer #2: (No Response)

Reviewer #3: All comments have been addressed

2. Is the manuscript technically sound, and do the data support the conclusions?

The manuscript must describe a technically sound piece of scientific research with data that supports the conclusions. Experiments must have been conducted rigorously, with appropriate controls, replication, and sample sizes. The conclusions must be drawn appropriately based on the data presented.

Reviewer #1: (No Response)

Reviewer #2: Yes

Reviewer #3: Yes

3. Has the statistical analysis been performed appropriately and rigorously?

Reviewer #1: (No Response)

Reviewer #2: No

Reviewer #3: Yes

4. Have the authors made all data underlying the findings in their manuscript fully available?

The [PLOS Data policy](http://www.plosone.org/static/policies.action#sharing) requires authors to make all data underlying the findings described in their manuscript fully available without restriction, with rare exception (please refer to the Data Availability Statement in the manuscript PDF file). The data should be provided as part of the manuscript or its supporting information, or deposited to a public repository. For example, in addition to summary statistics, the data points behind means, medians and variance measures should be available. If there are restrictions on publicly sharing data—e.g. participant privacy or use of data from a third party—those must be specified.

Reviewer #1: (No Response)

Reviewer #2: Yes

Reviewer #3: Yes

5. Is the manuscript presented in an intelligible fashion and written in standard English?

PLOS ONE does not copyedit accepted manuscripts, so the language in submitted articles must be clear, correct, and unambiguous. Any typographical or grammatical errors should be corrected at revision, so please note any specific errors here.

Reviewer #1: (No Response)

Reviewer #2: Yes

Reviewer #3: Yes

6. Review Comments to the Author

Please use the space provided to explain your answers to the questions above. You may also include additional comments for the author, including concerns about dual publication, research ethics, or publication ethics. (Please upload your review as an attachment if it exceeds 20,000 characters)

Reviewer #1: (No Response)

Reviewer #2: Thanks for your detailed modification, and there exists minor questions as following:
1、 Why chose Kruskal–Wallis non-parametric test for continuous variables, instead of ANOVA? Were all continuous variables skewness distribution?

**Response**

Thank you for drawing our attention to this point.

We checked the distribution of all continuous variables and confirmed that the data for several variables follow a normal distribution; these data were then reanalyzed using ANOVA.

We have accordingly changed the sentence on lines 162–164 (page 7) to read, ‘Continuous variables, expressed as means and standard deviations or medians and interquartile ranges, were compared by means of analysis of variance or the Kruskal–Wallis non-parametric test.’

2、 In Table 1, the P value between any two comparisons should be marked, especially when compared to NA group.

**Response**

Thank you for your comment.

In addition to using ANOVA or the Kruskal–Wallis test to find significant differences among the *NA*, *Disability*, and *Had died* groups, we used the Tukey or Steel–Dwass test to compare data for the *NA* group versus the *Disability* group and the *NA* group versus the *Had died* group.

This is now stated on lines 165 and 166 (page 7) (‘We also used the Tukey test or Steel–Dwass test to compare data for the continuous variables between the groups.’), and the statistical package is named on lines 193 and 194 (page 8) (‘Additionally, we used R version 4.1.2 (the R Foundation for Statistical Computing, Vienna, Austria) software for the Steel–Dwass test.’). Relevant p values and explanatory footnotes have been added to Table 1.

3、 In Table 2, since urinary NAG/creatinine ratio quartile is a ranked variable, P-trend value and the P value for Q2\Q3\Q4 compared to Q1 should be listed. Therefore, as for ranked variable, Pearson chi-square test is not suitable for categorical variables to estimate P values.

**Response**

Thank you for making this important point.

We used the logistic regression model, instead of the Pearson chi-square test, to estimate differences among quartiles. Furthermore, we calculated p values for Q2, Q3, or Q4 versus Q1; significant differences are highlighted with asterisks in Table 2.

Use of the logistic regression model is now mentioned in the sentence on lines 164 and 165 (page 7) (‘Categorical variables, expressed as proportions, were compared using the chi-square test or the logistic regression model.’) and in Table 2, footnote c.

Reviewer #3: (No Response)

7. PLOS authors have the option to publish the peer review history of their article ([what does this mean?](https://journals.plos.org/plosone/s/editorial-and-peer-review-process#loc-peer-review-history)). If published, this will include your full peer review and any attached files.


If you choose “no”, your identity will remain anonymous but your review may still be made public.


**Do you want your identity to be public for this peer review?** For information about this choice, including consent withdrawal, please see our [Privacy Policy](https://www.plos.org/privacy-policy).

Reviewer #1: No

Reviewer #2: No

Reviewer #3: No

[NOTE: If reviewer comments were submitted as an attachment file, they will be attached to this email and accessible via the submission site. Please log into your account, locate the manuscript record, and check for the action link "View Attachments". If this link does not appear, there are no attachment files.]

While revising your submission, please upload your figure files to the Preflight Analysis and Conversion Engine (PACE) digital diagnostic tool, <https://pacev2.apexcovantage.com/>. PACE helps ensure that figures meet PLOS requirements. To use PACE, you must first register as a user. Registration is free. Then, login and navigate to the UPLOAD tab, where you will find detailed instructions on how to use the tool. If you encounter any issues or have any questions when using PACE, please email PLOS at [figures@plos.org](mailto:figures@plos.org). Please note that Supporting Information files do not need this step.
